# Biotic and Environmental Drivers of Plant Microbiomes Across a Permafrost Thaw Gradient

**DOI:** 10.3389/fmicb.2020.00796

**Published:** 2020-05-15

**Authors:** Moira Hough, Amelia McClure, Benjamin Bolduc, Ellen Dorrepaal, Scott Saleska, Vanja Klepac-Ceraj, Virginia Rich

**Affiliations:** ^1^Department of Ecology and Evolutionary Biology, University of Arizona, Tucson, AZ, United States; ^2^Department of Biological Sciences, Wellesley College, Wellesley, MA, United States; ^3^Department of Microbiology, College of Arts and Sciences, The Ohio State University, Columbus, OH, United States; ^4^Climate Impacts Research Centre, Umeå University, Umeå, Sweden

**Keywords:** microbial community assembly, permafrost thaw, plant–microbial interactions, keystone species, climate feedbacks, IsoGenie Project, Stordalen Mire

## Abstract

Plant-associated microbiomes are structured by environmental conditions and plant associates, both of which are being altered by climate change. The future structure of plant microbiomes will depend on the, largely unknown, relative importance of each. This uncertainty is particularly relevant for arctic peatlands, which are undergoing large shifts in plant communities and soil microbiomes as permafrost thaws, and are potentially appreciable sources of climate change feedbacks due to their soil carbon (C) storage. We characterized phyllosphere and rhizosphere microbiomes of six plant species, and bulk peat, across a permafrost thaw progression (from intact permafrost, to partially- and fully-thawed stages) via 16S rRNA gene amplicon sequencing. We tested the hypothesis that the relative influence of biotic versus environmental filtering (the role of plant species versus thaw-defined habitat) in structuring microbial communities would differ among phyllosphere, rhizosphere, and bulk peat. Using both abundance- and phylogenetic-based approaches, we found that phyllosphere microbial composition was more strongly explained by plant associate, with little influence of habitat, whereas in the rhizosphere, plant and habitat had similar influence. Network-based community analyses showed that keystone taxa exhibited similar patterns with stronger responses to drivers. However, plant associates appeared to have a larger influence on organisms belonging to families associated with methane-cycling than the bulk community. Putative methanogens were more strongly influenced by plant than habitat in the rhizosphere, and in the phyllosphere putative methanotrophs were more strongly influenced by plant than was the community at large. We conclude that biotic effects can be stronger than environmental filtering, but their relative importance varies among microbial groups. For most microbes in this system, biotic filtering was stronger aboveground than belowground. However, for putative methane-cyclers, plant associations have a stronger influence on community composition than environment despite major hydrological changes with thaw. This suggests that plant successional dynamics may be as important as hydrological changes in determining microbial relevance to C-cycling climate feedbacks. By partitioning the degree that plant versus environmental filtering drives microbiome composition and function we can improve our ability to predict the consequences of warming for C-cycling in other arctic areas undergoing similar permafrost thaw transitions.

## Introduction

As ecosystems warm and undergo transitions due to climate change, we are seeing significant shifts in microbial community ecology and function with important implications for carbon (C) storage (Zhou et al., 2012). This is especially true in arctic peatlands which are warming fast and undergoing major ecosystem transitions as permafrost thaws (McCalley et al., 2014; Mondav et al., 2017; Monteux et al., 2018). Microbial communities are strongly impacted by the environmental changes associated with permafrost thaw, particularly hydrology, available C-substrates, and plant communities. It has been well established that different stages of permafrost-thaw harbor substantially different bulk peat microbial communities, and these changes have major implications for C-cycling, particularly methane (Tveit et al., 2013; McCalley et al., 2014; Mondav et al., 2017; Monteux et al., 2018; Woodcroft et al., 2018a). However, plant-associated microbial communities remain under-studied. In particular, it is unclear whether plant microbiome changes are determined more by environmental changes as permafrost thaws, or by the biotic effects of plant community changes especially in the rhizosphere (Tkacz et al., 2015) and phyllosphere (Redford et al., 2010). Abiotic effects are often thought of as operating at a macro-scale as a filter that selects a potential species pool, upon which biotic effects act at the micro-scale (Aguilar et al., 2017; Cadotte and Tucker, 2017; Thakur and Wright, 2017). However, in the case of a permafrost-thaw front, both effects take place on a similar scale, with partial overlap of plant species (and therefore biotic effects) across a strong but geographically small environmental gradient. This raises the question of whether microbial community composition is more strongly determined by plant associate (i.e., biotic filtering) or thaw stage (i.e., environmental filtering), and whether plant selection on the microbial community has implications for C-cycling. This is especially important because permafrost is estimated to contain more than a third of the C in the top 1–3 m of the Earth’s soil and its thaw is expected to lead to C releases to the atmosphere in the range of 37–174 Pg by 2,100 which would increase climate warming by 0.13–0.27°C (Tarnocai et al., 2009; Hugelius et al., 2014; Schuur et al., 2015). Microbes serve as the primary decomposers and gatekeepers determining how much C will remain in the ecosystem versus being released to the atmosphere as CO_2_ and CH_4_. Indeed, in some systems changes in microbial community structure and function have been shown to mitigate C-release predicted due to warming alone (Zhou et al., 2012). Therefore accurate prediction of the magnitude of C releases from the arctic requires understanding how microbial communities will be impacted by permafrost thaw.

While thawing permafrost peat microbial communities have been well studied (Tveit et al., 2013; McCalley et al., 2014; Mondav et al., 2017; Monteux et al., 2018; Woodcroft et al., 2018b), the plant-associated microbial communities in these systems have not been well characterized, nor is it clear to what extent plant associations are responsible for the changes in bulk peat microbial community. In most systems, rhizosphere and phyllosphere microbial communities seem to draw at least some members from the communities present in the surrounding environment but they form distinct communities based on factors such as the species, genotype, and health of the host-plant as well as the compartment in question (Delmotte et al., 2009; Redford et al., 2010; Fonseca et al., 2016; Nuccio et al., 2016). For instance, the phyllosphere is likely to be enriched in alpha-proteobacteria, particularly those adapted to stress and with either one-C central metabolism (e.g., methanotrophy and methylotrophy) such as *Methylobacterium*, or a diverse range of substrate use such as *Sphingomonas* (Delmotte et al., 2009; Knief et al., 2012). Since both metabolic types have been found on the same phyllosphere samples, this divergence in metabolic strategy may indicate that some organisms capitalize on plant-associated substrates whereas others remain generalists in a resource-limited environment. Controls on rhizosphere community structure are complex, including the structure of the roots, the density, size of their filaments, and on fungal associations of the host plant (Parniske, 2008). These rhizosphere-associated communities are often less diverse than the microbial communities in the surrounding soil matrix but more diverse than the phyllosphere (Berg and Smalla, 2009; Knief et al., 2012). Rhizosphere communities are often enriched in alpha- and beta-proteobacteria, and depleted in Actinobacteria compared to soil microbial communities, indicating plant influence on community structure and composition (Knief et al., 2012; Nuccio et al., 2016).

Plant selection on leaf and root microbiomes is potentially important in mediating microbial controls on decomposition (Strickland et al., 2009) and ultimately C flux to the atmosphere. The first communities that have the opportunity to decompose plant material are the microbes colonizing the plant itself (Tláskal et al., 2016). Plants often have specialized phyllosphere communities which accompany senescing leaf tissue into the soil (Redford et al., 2010; Nissinen et al., 2012; Bragina et al., 2015; Lebeis, 2015; Tláskal et al., 2016; Baldrian, 2017). Members of the phyllosphere have been shown to participate in the initial decomposition of fresh organic material before being replaced by microbes from the bulk soil community, and leaf litter composition can influence decomposition dynamics such as fungal to bacterial ratios (Kögel-Knabner, 2002; Thiessen et al., 2013; Tláskal et al., 2016; Baldrian, 2017). Additionally, plant root exudates are known to be a source of C to soil and to determine rhizosphere microbial community composition (Tkacz et al., 2015). Microbial response to differences in C quality in root exudates (such as through shifts in activity or bacterial to fungal ratios) can influence decomposition dynamics such as by stimulating decomposition or causing additional C release from bulk peat through priming effects (Cheng et al., 2014; Pegoraro et al., 2019). Permafrost thaw has been shown to dramatically alter root growth patterns including quantity and quality of litter inputs which can be expected to influence microbial community composition (Blume-Werry et al., 2019). However, research characterizing permafrost and bulk peat microbial communities in arctic systems (Mackelprang et al., 2011; Hultman et al., 2015; Mondav et al., 2017; Woodcroft et al., 2018a), has yet to clearly describe the role of plants in influencing the microbial community composition, decomposition, and C cycling.

Since permafrost thaw results in changes to both environmental and plant filtering effects on the microbial community, we investigated their relative importance in structuring rhizosphere versus phyllosphere microbial communities, at a well-studied permafrost thaw gradient in Stordalen Mire, Sweden. In this area, as permafrost thaws, palsa peat mounds (which rise above the surrounding wet areas due to a supportive core of ice) collapse to form waterlogged bog areas which initially remain hydrologically disconnected from the later thaw stages but eventually sink and form fully inundated, hydrologically connected, nutrient-rich fens. As these three soil environments differ more strongly than the air, we hypothesized that biotic filtering would be more important than environmental filtering in the phyllosphere, but the reverse would be true in the rhizosphere. Microbial communities are highly diverse and often include taxa which may be more important to community structure than expected based on abundance measures (keystone species) as well as many taxa that are of little importance to community structure. Therefore, we consider two metrics by which microbial community differences may be measured across sites, compartments (rhizosphere, phyllosphere, or peat), and plant associates: (1) differences in relative abundance and (2) differences in importance to network structure (or keystoneness). Furthermore, to investigate whether these community composition differences could be important to the ecosystem’s C-cycling, we investigated functional groups important to CH_4_ cycling (as a particularly important C-cycling function which is also phylogenetically constrained). This approach allows us to identify the degree to which plant and environmental filtering influence C cycling through changes in microbial functional groups as well as overall microbial community composition, structure, and putative function.

## Materials and Methods

### Site and Sample Collection

**FIGURE 1:**
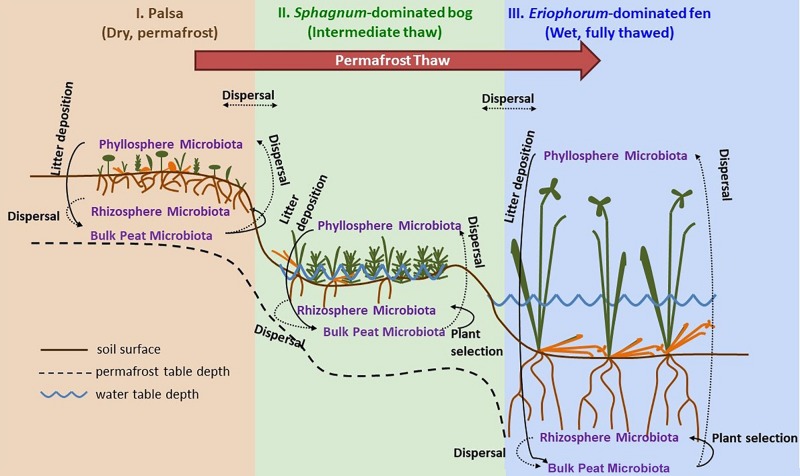
Permafrost thaw progression showing directions of possible microbial dispersal in the context of changes in plant community composition and hydrology as ecosystems transition from: I. permafrost-underlain palsa, to II. partially thawed bog, to III. fully thawed and inundated fen. The underlying thaw progression figure has been adopted from Johansson et al. (2006) and modified substantially.

Samples were collected at three time points through the 2015 growing season: early season (20 and 21 June), peak growing season (20 and 23 July), and late growing season (2 September). Stordalen Mire is located 10 km east of Abisko, at 68° 219 N, 18° 499 E, and is 363 m above sea level (with ecologically relevant microtopography across the Mire spanning several meters’ elevation). The site is managed by the Abisko Scientific Research Station, the University of Stockholm, and the Integrated Carbon Observation System. Within the mire, there are three main habitats spanning a permafrost thaw gradient (Figure 1): no thaw (palsa type), initial partial thaw (bog type), and complete thaw (fen type). Palsas consist of raised, permafrost underlain areas characterized by low shrubs (e.g., *Betula nana, Empetrum nigrum, Andromeda polifolia, Vaccinium* spp.), forbs (e.g., *Rubus chamaemorus*), graminoids (e.g., *Eriophorum vaginatum*), lichens, and drier mosses (e.g., *Sphagnum fuscum*). Bogs are wetter low-lying areas often still underlain by permafrost lenses characterized by more hydric species of sphagnum (e.g., *Sphagnum balticum*) and small sedges (e.g., *E. vaginatum* and *Carex rotundata*). Fens are formed after complete permafrost thaw and collapse. They are characterized by standing water, and hydric sphagnum and sedge species (e.g., *Eriphorum angustifolium*, *C. rotundata*, and *Carex rostrata*). As of 2010, 49% of the area within Stordalen was made up of intact palsa, 37% was made up of partially thawed bog, 12% was made up of fully thawed fen (Bäckstrand et al., 2010). From 1970 to 2000, palsa extent shrank while bog sites expanded by 3% and fen sites expanding by 54% (Bäckstrand et al., 2010).

Three areas were selected where all three habitats could be found within close (20 m^2^) proximity. Samples of leaf, root, and associated peat for seven abundant, habitat-typical plant species were selected for phyllosphere and rhizosphere characterization across the mire’s three habitats. Species representative of palsa areas were: *R. chamaemorus*, *A. polifolia*, *E. vaginatum*, and *Sphagnum* sp. Species representative of bog areas were: *A. polifolia*, *E. vaginatum*, *C. rotundata*, and *Sphagnum* sp. Species representative of fen areas were: *E. angustifolium*, and *C. rotundata*. Note that palsa and bog *Sphagnum* spp. differed, and that several species were representative in more than one habitat (and were sampled separately in each habitat in case of physiological differences). Fewer species were selected to represent the fen habitat based on its much lower plant diversity (Johansson et al., 2006). Replicate plants were sampled in two ways: spatially distributed and spatially proximal. For the former, one individual of each habitat-representative species was sampled at each of the three locations (generating three spatially distributed replicates). For the latter, at one location, two additional sets of plant samples were collected for each habitat-representative species (generating a total of three spatially proximal replicates). In addition, a single peat sample was taken at each location, in each habitat (for three spatially distributed replicates per habitat).

All material was collected using forceps sterilized with ethyl alcohol and rinsed twice with water. For each plant, phyllosphere samples were collected first, consisting of approximately 2 g of living *Sphagnum* spp. tissue, all leaf tissue from *A. polifolia* and *R. chamaemorus* plants, and 1 cm^2^ of sedge plants (*Eriophorum* spp. and *C. rotundata*). When all phyllosphere sample material had been collected, a serrated edge knife was used to cut around the base of the plant. The plant was then pulled out with associated peat still attached. “Bulk peat” samples were collected as 15 mL of peat from 2 to 3 cm deep, from as close to the root as possible without touching the root. Rhizosphere samples were collected by snipping small pieces of the root fibers into a 1.5 mL Eppendorf tube. Both the primary root and lateral roots were included and the root tip was excluded. A total of 4 cm of main and lateral roots were sampled from *A. polifolia* and *R. chamaemorus* plants, and one segment of the main root node and 2–4 fine root hairs were sampled for each sedge rhizosphere sample. While *Sphagnum* spp. plants do not have roots, 2 g samples were taken from the base of the stalk where the tissue was brown but still attached to the living stalk. After collection, the samples were stored in a cooler, and then transferred to a −80°C freezer within 4 h of collection. The samples were shipped on dry ice to Wellesley College and immediately stored at −80°C until further processing.

### DNA Extraction and Sequencing of 16S rRNA Gene

For each sample a standardized quantity of 50 mg of *Sphagnum* spp. photosynthetic and non-photosynthetic tissue, 75 mg of angiosperm leaf tissue, 100 mg of angiosperm root material, or 50 mg of peat was used for DNA extraction. In all cases bulk samples were used, meaning that results include both endo- and epiphytic microbial communities. Total DNA from each sample was extracted using the PowerSoil^®^ DNA Isolation Kit (MoBio, Carlsbad, CA, United States) according to the manufacturer’s instructions and eluted in 50 μL of elution buffer. After extraction, DNA was quantified using NanoDrop (Thermo Scientific, Inc., Wilmington, DE, United States) and stored at −20°C.

Sequencing at Argonne National Laboratories followed the protocols of Caporaso et al. (2012). PCR amplification of the V4 region of 16S rRNA gene made use of the forward primer 515F (5′-GTGCCAGCMGCCGCGGTAA-3′) and the reverse primer 806R (5′-GGACTACHVGGGTWTCTAAT-3′). PCR amplicons were quantified by PicoGreen (Invitrogen, Carlsbad, CA, United States) using a plate reader, and amplicons were pooled in equal concentrations into a single tube. This pool was cleaned up using UltraClean PCR Clean-Up Kit (MoBio, Carlsbad, CA, United States) and quantified using the Qubit (Invitrogen, Carlsbad, CA, United States). The pooled samples were sequenced on the Illumina MiSeq platform (Illumina, San Diego, CA, United States) according to the sequencing procedures described in Caporaso et al. (2012). Sequences are available in the National Center for Biotechnology Information Sequence Read Archive, Study Accession: PRJNA599435.

### Bioinformatic Analysis

An in-house workflow was used to process operational taxonomic unit (OTU) data, which was based primarily on Quantitative Insights into Microbial Ecology (Caporaso et al., 2010) and SparCC. Detailed information about the SparCC workflow can be found in Friedman and Alm (2012). Briefly, paired-end sequences were first joined, then split into libraries according to their index. Sequences were then filtered according to quality using USEARCH fastq_filter (-fastq_maxee 0.5), dereplicated and screened against phiX174. Surviving sequences were clustered into OTUs at 97% average nucleotide identity, then taxonomy assigned using the Greengenes database (DeSantis et al., 2006) version 13_8 using the Ribosomal Data Project classifier (McDonald et al., 2012). Finally, OTUs matching chloroplast and mitochondrial sequences were removed from the analysis. Unifrac analysis was performed in QIIME (Lozupone and Knight, 2005). OTU tables were pruned to address specific experimental questions, then LEfSe biomarker analysis was completed (Segata et al., 2011).

Network analysis began with filtering of OTU tables to exclude any OTUs with fewer than 10 counts and represented in fewer than three total samples across all habitat, tissue or plant types. To generate networks, OTU tables were split according to habitat, tissue type, and plant type, and mean OTU abundances for each sample type were calculated based on filtered tables of counts as described above. Compositionality-robust correlations of each OTU table (15 total) was generated using SparCC using the median of 20 iterations. Data were resampled and bootstrapped 100 times (20 iterations) to generate correlation *p*-values. Correlation matrices were then processed using python packages Numpy (Walt et al., 2011), Scipy (Virtanen et al., 2020), and Pandas (McKinney, 2010). Each correlation matrix has its correlations filtered according to its *p*-value (<0.05), and networks were built using the filtered correlations as an adjacency matrix and rendered using python-igraph (Csardi and Nepusz, 2006) with visual refinement using Adobe Illustrator CC such that OTUs served as “nodes” or vertices, and absolute correlation values as the “edges” between OTUs.

We interpreted OTU PageRank score, which quantifies connectivity, as a metric of ecological importance. We considered the most important OTUs to be those in the 99th percentile for each network based on PageRank scores, and compared interpretation based on PageRank to the additional network metrics of degree, transitivity, betweenness, and closeness. Demonstration of the strong correlation of PageRank with the “consequentiality” of nodes in diverse settings: for identification of key metabolic genes in pathways of co-occurrence gene networks (Browne et al., 2016; Wang et al., 2017, extinction cascades in food webs (Allesina and Pascual, 2009), and complex phylogenetic signals in communities of co-occurring bacteria (Estrada-Pena et al., 2018). This metric has been successfully applied to extinction food webs (Allesina and Pascual, 2009). The nature of the metric (which quantifies the connections of a node to others, with weighting for those neighbors’ respective connectivity too), and these other studies, supported its use as an indicator of keystone organisms. In order to relate OTU importance and abundance (which are on different scales), we used the rank of each within every sample, rather than its absolute value.

Microbial functional groups of interest were methanogens and methanotrophs. While functional assignation of organisms identified using 16S sequencing cannot be definitive, methane cycling traits are generally highly conserved (Martiny et al., 2015) meaning that organisms belonging to taxonomic groups known to produce or consume methane are likely to have similar lifestyles. Methanogens were identified based on classification into one of the following orders known to be dominated by methanogens and active at our site: Methanosarcinales, Methanobacteriales, Methanomicrobiales (Mondav et al., 2014; Woodcroft et al., 2018b; Evans et al., 2019). Methanotrophs were identified based on classification into one of the following groups known to be predominantly methanotrophic and active at our site: order Methylacidiphilales, families Beijerinckiaceae, Hyphomicrobiaceae, Methylocystaceae, Crenotrichaceae, and Methylococcaceae (Tveit et al., 2013; Knief, 2015; Singleton et al., 2018; Smith and Wrighton, 2019).

### Statistical Analysis

NMDS and variance partitioning were performed on both Unifrac and Bray–Curtis distances of OTU tables for all samples. Significance tests were performed with redundancy analysis for Unifrac data and distance-based redundancy analysis for Bray–Curtis distances using the vegan package in R (Oksanen et al., 2019). Hierarchical clustering (based on Jaccard and Bray–Curtis distances) was performed on both abundance and importance scores of OTUs in networks. All statistical analyses were performed in R studio running R version 3.6.0 (R Studio Team, 2018; R Core Team, 2019).

## Results

A total of 350 samples were sequenced and rarefied to include 1,000 high-quality bacterial or archaeal 16S rRNA gene sequences per sample. Alpha diversity varied between rarefaction analysis indicating that full sampling depth was achieved only for samples with lower levels of diversity, and not with samples from the fully thawed fen peat (Supplementary Figure S1). Out of all sequences, 99.6% were assigned to Bacteria and 0.4% to Archaea – the latter is consistent with previous work from this site (Mondav et al., 2017) and reflects the known biases of the V4 primer set against Archaea (Parada et al., 2016). Across all samples, there were a total of 373 unique OTUs (1,436 in the unrarefied dataset) which were assigned to 170 genera and 31 known phyla (170 genera and 32 phyla in the unrarefied dataset). Diversity within a sample ranged from 124 to 361 OTUs (181 to 609 in the unrarefied dataset). The four most dominant phyla when considering the entire dataset were Proteobacteria (44%), Acidobacteria (22.9%), Bacteroidetes (8.9%), and Verrucomicrobia (7.8%). This profile matched the general profiles found in previous investigation into bulk peat communities at this site by Mondav et al. (2017) but with stronger and more consistent dominance of Proteobacteria and lower relative abundance of Acidobacteria across all habitat types, particularly in the phyllosphere. This is generally in line with the findings of Mondav et al. (2017) despite major differences in methodology.

### Diversity and Composition of Bacterial Taxa Across Sample Types

**FIGURE 2:**
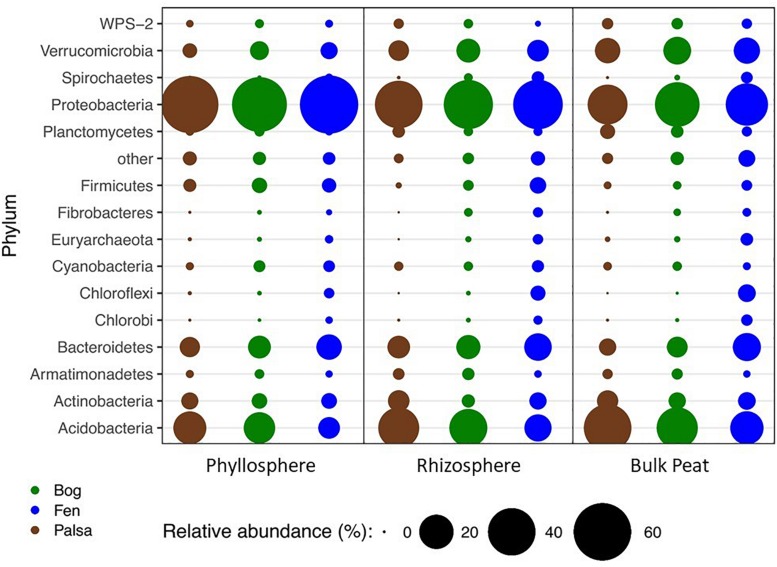
Changes in relative abundance of bacterial phyla across habitats (palsa = brown, bog = green, and fen = blue) and compartments (phyllosphere, rhizosphere, and bulk peat). Circle size corresponds to percent relative abundance.

A total of 66 OTUs were ubiquitous across all sample types (habitat, compartment, and plant) after removal of chloroplast sequences. These belonged to 10 phyla: Acidobacteria, Actinobacteria, Armatimonadetes, Bacteroidetes, Chlamydiae, Cyanobacteria, Planctomycetes, Proteobacteria, Verrucomicrobia, and WPS-2. Bacterial composition at the phylum level showed the same major phyla represented across the permafrost thaw gradient with variation in relative abundance by habitat type and compartment (Figure 2). Based on LEfSe analysis, we identified phyla that were enriched in abundance in one habitat as compared to the others within each compartment (LDA > 2). The palsa was enriched in Acidobacteria and Planctomycetes in all three compartments, as well as Actinobacteria and Chlamydiae in the rhizosphere and peat, and WPS-2 and FCPU426 in the rhizosphere only. The bog was enriched in WS4 in all compartments, TM6 in the phyllosphere and rhizosphere, Armatimonadetes and Verrucomicrobia in the rhizosphere and peat, OD1 in the phyllosphere only, FBP and Proteobacteria in the rhizosphere only, and WPS-2 in the peat only. The fen contained the greatest number of enriched phyla with Bacteroidetes, Chlorobi, Chloroflexi, Fibrobacteres, Firmicutes, Nitrospirae, Spirochaetes, Euryarchaeota, and Parvarchaeota enriched in all three compartments. The fen rhizosphere and peat were also enriched in Crenarchaeota, Elusimicrobia, OP3, and WWE1, with the rhizosphere enriched for OD1, TM7, AD3, Cyanobacteria, and Fusobacteria, and the peat enriched in Gemmatimonadetes (Supplementary Figure S2).

**FIGURE 3:**
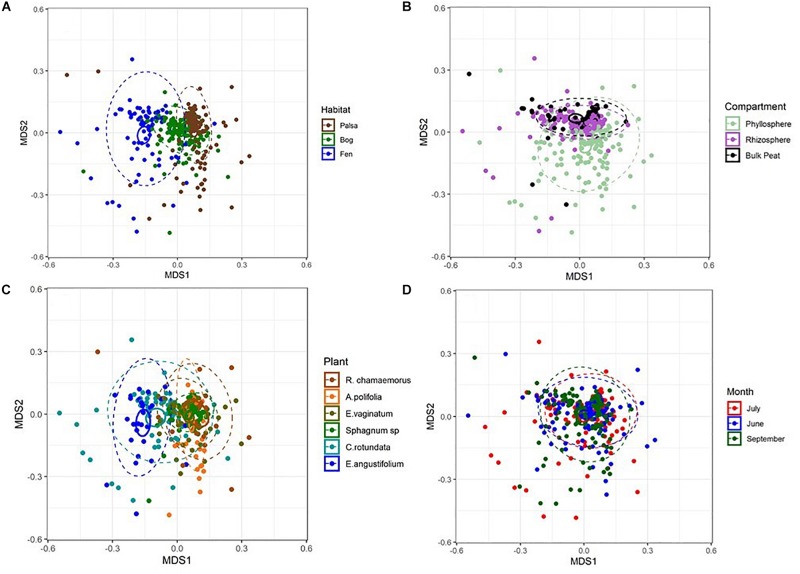
NMDS plots of Bray–Curtis distances between samples from each **(A)** habitat, **(B)** compartment, **(C)** plant associate, and **(D)** month. Outer ellipses include 95% of points for a habitat, inner ellipses are 95% confidence around the centroid. Stress = 0.17.

NMDS based on Bray–Curtis distances between OTUs showed separation of centroids by habitat with only partial separation by compartment and plant type and very little separation by season (Figure 3). PERMANOVA with plant and habitat nested within compartment indicated significant differences (*p* < 0.001) between communities across all factors (compartment, plant, habitat, and month) as well as significant interaction terms (*p* < 0.001). Variance partitioning indicated that when considered individually, habitat and plant accounted for the majority of the variation with compartment playing a smaller role and sample month having almost no impact (Table 1). However, the most variance was explained when habitat, plant associate, and compartment were all considered (adjusted *r*^2^ = 0.27, *p* < 0.001). Repeating the analyses using phylogenetically weighted Unifrac values to take phylogenetic similarity into account provided similar results but with an increase in the unique amounts of variance explained, particularly by compartment (adjusted *r*^2^ = 0.39, *p* < 0.001, Table 1).

**TABLE 1:** Variance explained by each factor based on Bray–Curtis and Unifrac distances of total dataset and Bray–Curtis distances of only OTUs included in networks.

	All OTUs		OTUs in Networks	Keystone OTUs
Distance metric	*Bray–Curtis*	*Unifrac*	*Bray–Curtis*	*Bray–Curtis*
Compartment total *R*^2^	0.07*	0.12*	0.07*	0.07*
Unique *R*^2^	0.07*	0.11*	0.07*	0.07*
Plant total *R*^2^	0.16*	0.23*	0.16*	0.19*
Unique *R*^2^	0.04*	0.04*	0.04*	0.04*
Habitat total *R*^2^	0.15*	0.24*	0.16*	0.19*
Unique *R*^2^	0.04*	0.05*	0.04*	0.00*
Month total *R*^2^	0.01*	0.00	0.00*	0.00
Unique *R*^2^	0.01	0.00	0.00	0.00
All	0.27*	0.39*	0.30*	0.33*

*Total *R*^2^ is variance explained by that factor including fractions that overlap with variance explained by other factors. Unique *R*^2^ is only the variance explained by a factor that does not overlap with any other factor. All *R*^2^ values are adjusted for multiple tests. Starred values are significant at *p* < 0.001 based on redundancy analysis. Unique *R*^2^ for month for Bray–Curtis distance based analysis was significant at *p* < 0.004 and for OTU’s used in networks was significant at *p* = 0.026. Month unique and total *R*^2^ were not significant (*p* > 0.1) for Unifrac based analyses. Month total *R*^2^ was significant for keystone OTUs at *p* = 0.007 but unique *R*^2^ was not significant (*p* = 0.25).*

**FIGURE 4:**
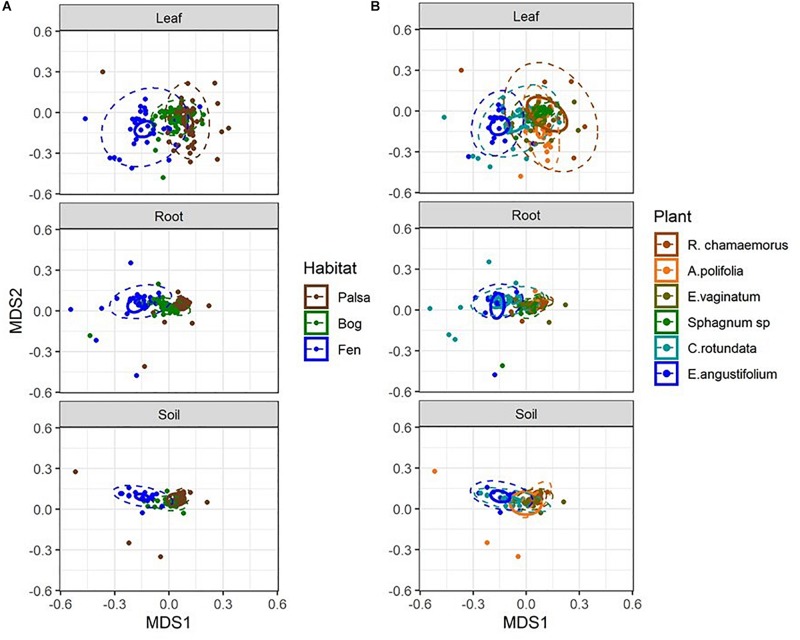
NMDS plots of Bray–Curtis distances separated by compartment and color-coded by **(A)** habitat type and **(B)** plant associate. Outer ellipses include 95% of points for a group, inner ellipses are 95% confidence around the centroid. Stress = 0.17.

Considering each compartment separately, NMDS plots indicated some separation by habitat type and plant associate, especially for phyllosphere and rhizosphere samples (Figure 4). Variance partitioning based on Bray–Curtis distance between communities showed different patterns for each compartment. In the rhizosphere, plant associate explained a similar to the amount of variance as habitat, but in the phyllosphere plant associate explained substantially more variance than habitat (Table 2). Plant associate explained a similar amount of variance in both rhizosphere and phyllosphere. However, habitat explained substantially more variance in the rhizosphere and the bulk peat than the phyllosphere. The total amount of variance explained by plant and habitat together was much higher in the rhizosphere and bulk peat than in the phyllosphere (Table 2). Much of the explained variance overlapped between both habitat and plant type, likely because plant species distribution was not even across all habitats.

**TABLE 2:** Variance partitioning by compartment based on Bray–Curtis distance between communities considering each compartment (phyllosphere, rhizosphere, and bulk peat) separately.

	Phyllosphere		Rhizosphere		Bulk Peat	
No. observations	142		144		76	
	All OTUs	Keystone	All OTUs	Keystone	All OTUs	Keystone
Plant total *R*^2^	0.17*	0.21*	0.27*	0.31*	0.23*	0.25*
Unique *R*^2^	0.08*	0.09*	0.08*	0.09*	–0.01	–0.01
Habitat total *R*^2^	0.11*	0.14*	0.26*	0.30*	0.33*	0.37*
Unique *R*^2^	0.03*	0.02*	0.07*	0.07*	0.10*	0.11*
All	0.20*	0.23*	0.35*	0.39*	0.33*	0.36*

*Unique *R*^2^ is only the variance explained by a factor that does not overlap with any other factor. All *R*^2^ values are adjusted for multiple tests. Starred values are significant at *p* < 0.001 based on redundancy analysis. Plant total *R*^2^ was not significant for bulk peat samples based on all OTUs (*p* = 0.827) or keystone taxa (*p* = 0.795).*

### Methane-Cycling Functional Groups

**FIGURE 5:**
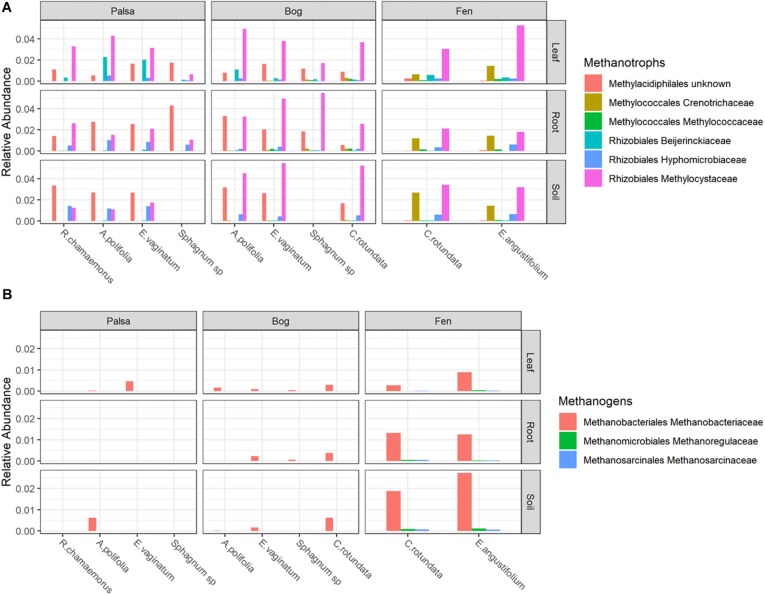
Relative abundances of **(A)** Methanotrophs and **(B)** Methanogens, grouped by habitat and compartment. Colors indicate plant associates.

Putative methanotrophs were identified as bacteria belonging to groups which have previously been shown to have active methanotrophic representatives at this site based on metagenomic and metatranscriptomic data (Tveit et al., 2013; Knief, 2015; Singleton et al., 2018; Smith and Wrighton, 2019). These were order Methylacidiphilales and families: Beijerinckiaceae, Hyphomicrobiaceae, Methylocystaceae, Crenotrichaceae, and Methylococcaceae. Methanotrophs, particularly members of Methylocystaceae, were found across all habitats, compartments, and plants though in varying abundances (Figure 5A and Supplementary Table S3). Members of Methylacidiphilales were common in palsa and bog habitats but rare in the fen, whereas members of Methylococcales were common in the fen but rare in the bog and not seen in the palsa. Variance partitioning on putative methanotrophs showed similar patterns to the full dataset except that habitat explained less and plant associate more of the variance in phyllosphere putative methanotroph distribution than in the full dataset (Table 3).

**TABLE 3:** Variance partitioning of putative methanotrophs by compartment based on Bray–Curtis distance between communities considering each compartment (phyllosphere, rhizosphere, and bulk peat) separately.

	Phyllosphere	Rhizosphere	Bulk Peat
No. observations	135	141	75
Plant total *R*^2^	0.18*	0.26*	0.21*
Unique *R*^2^	0.11*	0.07*	–0.01
Habitat total *R*^2^	0.08	0.28*	0.32*
Unique *R*^2^	0.01*	0.09*	0.10*
All	0.19*	0.36*	0.31*

*Unique *R*^2^ is only the variance explained by a factor that does not overlap with any other factor. All *R*^2^ values are adjusted for multiple tests. Starred values are significant at *p* < 0.001 based on redundancy analysis. Plant unique *R*^2^ less significant (*p* = 0.017) for Phyllosphere and not significant for bulk peat samples (*p* = 0.87).*

Putative methanogens were defined as Euryarchaeota in orders Methanosarcinales, Methanomicrobiales, Methanococcales, Methanobacteriales, Methanopyrales, Methanocellales, Methanomassiliicoccales, and “*Candidatus* Methanophagales” (Evans et al., 2019). Methanogens were found across all habitats and compartments as well as all plants except *R. chamaemorus*. Members of the family Methanobacteriaceae reached the highest abundances, especially in the fen, and were the most widespread (Figure 5B and Supplementary Table S4). Variance partitioning based on relative abundances did not match the pattern seen in the overall dataset. None of the phyllosphere variance was well explained. In the rhizosphere, plant associate explained substantially more variance than habitat, though overlap was high (Table 4).

**TABLE 4:** Variance partitioning of putative methanogens by compartment based on Bray–Curtis distance between communities considering each compartment (phyllosphere, rhizosphere, and bulk peat) separately.

	Phyllosphere	Rhizosphere	Bulk Peat
No. observations	46	64	41
Plant total *R*^2^	–0.01	0.36*	0.24*
Unique *R*^2^	–0.05	0.15*	0.028
Habitat total *R*^2^	–0.02	0.22*	0.25*
Unique *R*^2^	–0.01	0.07*	0.04
All	–0.02	0.37*	0.28*

*Unique *R*^2^ is only the variance explained by a factor that does not overlap with any other factor. All *R*^2^ values are adjusted for multiple tests. Starred values are significant at *p* < 0.001 based on redundancy analysis. Phyllosphere overall model was not significant (*p* = 0.615), plant unique *R*^2^ not significant (*p* = 0.125) and habitat unique *R*^2^ significant at *p* = 0.039.*

### Network Analysis

**FIGURE 6:**
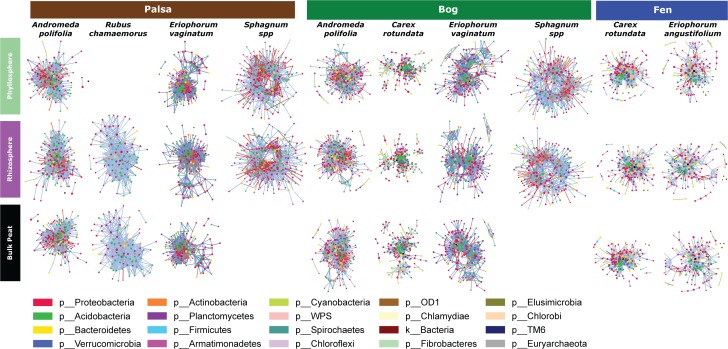
Network diagrams of the microbial community associated with each sample type (separated by habitat, compartment, and plant associate). Blue lines indicate positive correlations and red lines indicate negative correlations. Colors indicate phyla.

Because networks were based on the un-rarefied dataset, they contained greater diversity with a total of 1,436 OTUs. We avoided rarefication in order to preserve as many members of the community as possible; focusing on those whose importance was greater than their relative abundance. Rarefying data masks this signal. Since PageRank scores community members more highly when their surrounding community members are highly scored, rarefying even a single members’ abundance would diminish the importance of those to which it is connected. The mean diversity per network type was 361 with a minimum of 181 and a maximum of 609. Variance partitioning on the OTUs included in the networks recapitulated the results seen in the entire dataset with slight increases in the amount of variance explained by plant and habitat, especially when using only keystone OTUs (Table 1). Network structure varied substantially by plant associate, retaining some similarity across habitat types for a given plant species (Figure 6). *Sphagnum*, in particular, showed a characteristic “donut” structure regardless of habitat – despite these being different species in the different habitats. This structure reflects a lack of a central hub of OTUs, with instead dispersed interconnected hubs. This may indicate the presence of multiple sub-communities connected by a few interacting species, which could be caused by distinct niches. A similar structure was found in a few other networks such as *E. vaginatum* in the bog, but was not consistent across habitats. Similarities in network structure between tissue types of a given plant species are not meaningful since the shape of the network structure visualization was held constant for each plant species in each habitat so only node size and connectivity varied across tissue types.

**FIGURE 7:**
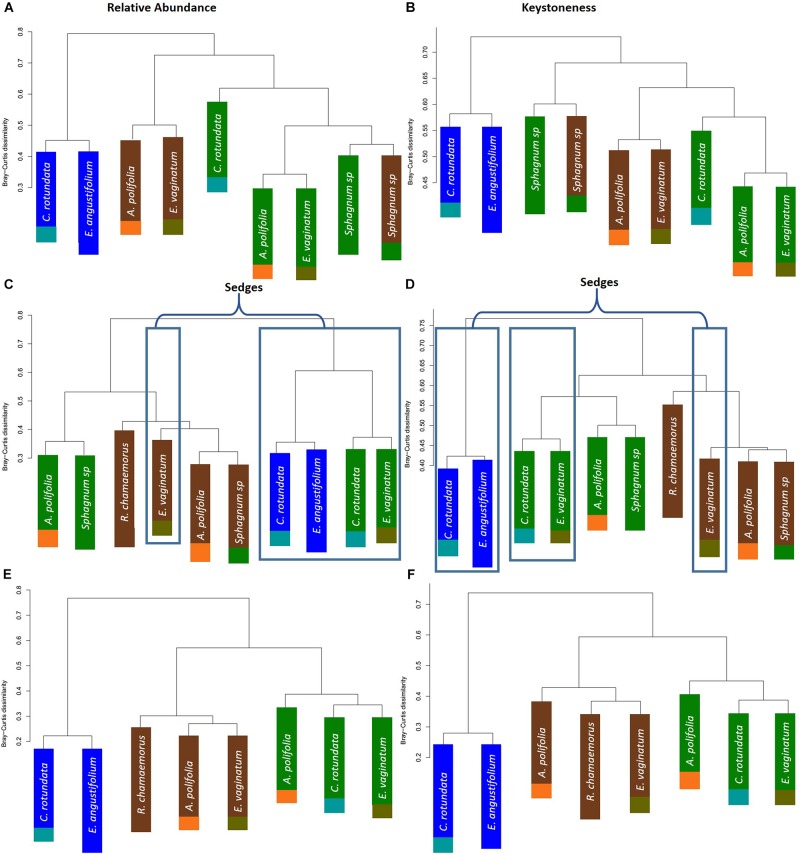
Hierarchical clustering of Bray–Curtis dissimilarity between community networks weighting organisms by **(A,C,E)** relative abundance for a given habitat/plant and **(B,D,F)** relative importance for a given habitat/plant. Main colors correspond to habitat with brown = palsa, green = bog, blue = fen. Accent colors represent plant species with orange = *A. polifolia*, brown = *R. chamaemorus*, olive = *E. vaginatum*, dark green = *Sphagnum*, teal = *C. rotundata*, dark blue = *E. angustifolium*.

To further test whether microbial communities were more similar when sampled from the same habitat versus the same plant associate, we used hierarchical clustering using Bray–Curtis distances based on both relative abundance and network importance score (PageRank or keystoneness). The phyllosphere microbial communities mostly clustered by habitat rather than plant type with the exception of *Sphagnum* which clustered on its own based on importance to network but with the bog plants based on relative abundance (Figures 7A,B). Rhizosphere microbial communities clustered primarily by habitat when importance to network was considered, however, when relative abundance was considered sedges from the bog clustered with the fen sedges rather than with the other bog plants (Figures 7C,D). As would be expected, bulk peat microbial communities clustered by habitat based on both relative abundance and importance to network (Figures 7E,F). Hierarchical clustering based on Jaccard dissimilarity which considers only presence/absence not relative abundance showed no differences in the structure of groupings but resulted in increased dissimilarity between groups (Supplementary Figure S3).

### Keystone Organisms

**FIGURE 8:**
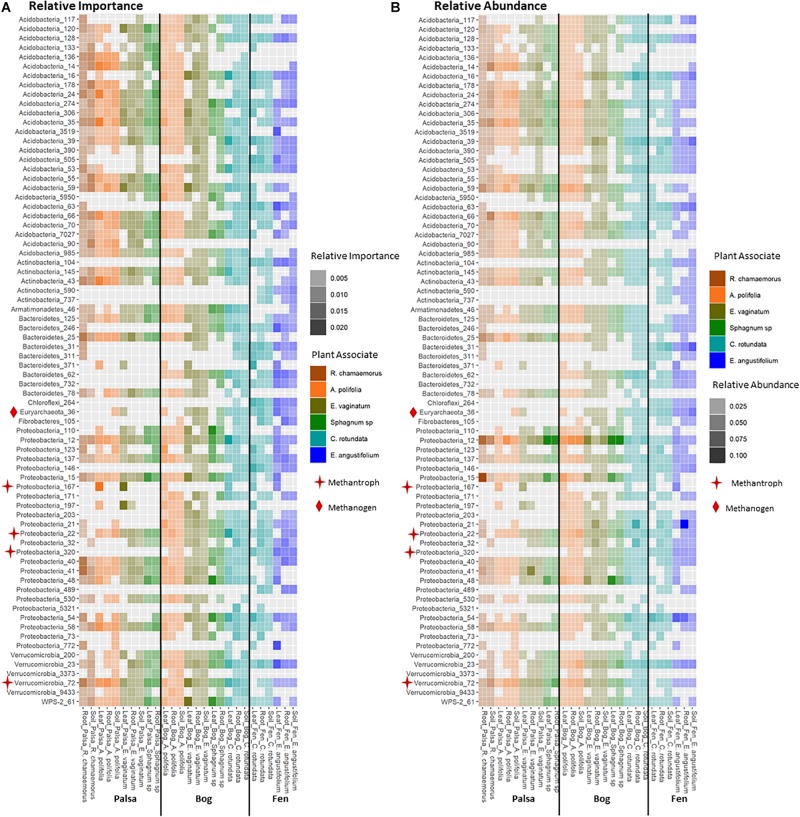
Comparison of **(A)** relative importance and **(B)** relative abundance of organisms in the 99th percentile of importance to network structure across habitat, compartments, and plant associates. Stars indicate methanotrophs and diamonds indicate methanogens.

Keystone organisms were defined as OTUs in the 99th percentile of importance for each network based on each organism’s PageRank score. PageRank has not typically been used as a metric for identification of keystone organisms in microbial communities. However, it has been shown to be strongly correlated with extinction cascades in food webs (Allesina and Pascual, 2009), identification of key metabolic genes in pathways of co-occurrence gene networks (Browne et al., 2016; Wang et al., 2017), and used to disentangle phylogenetic signals in communities of co-occurring bacteria (Estrada-Pena et al., 2018) thus indicating that it may indeed be a strong indicator of keystone organisms. PageRank score within a network was strongly correlated with betweenness centrality (Supplementary Figures S4, S5), which has previously been used as a metric for identifying keystone organisms (Martín González et al., 2010). Keystone OTUs also scored highly for closeness centrality, betweenness centrality, degree, and transitivity (Supplementary Figure S4). A total of 75 unique OTUs were identified as keystone organisms across the 27 different networks (Supplementary Table S1). This represented an average of four keystone taxa per network with a minimum of two and a maximum of seven (Supplementary Table S2). OTUs were generally only keystone for one or a few networks and none were keystones across all networks, habitats, compartments, or plants. Considering relative abundances of keystone OTUs across all samples indicated that they ranged from generalists (found across habitats, plants, and compartments) to specialists at any of the three levels (by habitat, plant type, and compartment) (Figure 8). Generalists included members of families Sinobacteraceae (OTU 12), Acetobacteraceae (OTU 137), and Acidobacteriaceae (OTU 274). Specialists included members of an unknown family in Chloroflexi (OTU 264) and Nakamurellaceae (OTU 737) found only in the fen, a member of an unknown family of Acidobacteria (OTU 90) found only in palsa, a member of Neisseriaceae (OTU 5321) found only in the rhizosphere, and members of Methylocystaceae (OTU 167) and Cytophagaceae (OTU 371) found only in phyllosphere. Variance partitioning based on relative abundances of the keystone organisms increased the total amount of variation explained to 33% but did not change the overall patterns in variance (Table 1). Six keystone OTUs belonged to functional groups of interest, one methanogen: OTU 36 (family Methanobacteriaceae) in the bog peat near a *Sphagnum* sp.; and five putative methanotrophs: OTU 22 (family Methylocystaceae) associated with *A. polifolia* rhizosphere in the bog and with peat near *C. rotundata* in the fen, OTU 40 (family Bradyrhizobiaceae) associated with the rhizosphere of *R. chamaemorus* in the palsa and *E. angustifolium* in the fen, OTU 72 (order Methylacidiphilales) associated with the rhizosphere of *A. polifolia* in the palsa and *C. rotundata* in the bog, OTU 167 (family Methylocystaceae) associated with the phyllosphere of *A. polifolia* and *E. vaginatum* in the palsa, OTU 171 (order Methylococcales) associated with the rhizosphere of *A. polifolia*, *E. vaginatum*, and the basal area of *Sphagnum* sp. in the bog, and OTU 320 (family Crenotrichaceae) associated with the bulk peat near *E. angustifolium* in the fen (Supplementary Table S1).

## Discussion

We investigated whether biotic filtering by plant associate could outweigh the importance of environmental filtering by habitat on microbial community composition across a permafrost thaw gradient. We found that the metric used for assessing differences between communities (importance to network versus relative abundance) has an impact on which taxa appear relevant. Under both approaches, we found evidence that in the rhizosphere, the environment first filters microbial community (especially through moisture), and then biotic interactions with plants further shape composition. In the phyllosphere, environmental conditions are less variable spatially so the effects of environmental filtering are less than the effects biotic influence on microbial community – though both are fairly small. However, we also found that the role of plant associate and habitat in shaping microbial communities may be very different for different microbial groups. Putative methanogens and methanotrophs appeared to be particularly strongly influenced by plant-associations, indicating that changes in plant community composition could have a significant influence on CH_4_ production from thawing permafrost ecosystems.

Below, we consider the following questions raised by our observations: What are the characteristics of the plant-associated microbiome as compared to the peat microbiome observed in other studies? Why did this study find relatively low influence of biotic filtering by plant associate on microbial community composition compared to other research? Does focusing analysis on keystone organisms that determine network structure provide clearer indication of the effects of filtering? Does plant community transition impact CH_4_ cycling in a way that contributes to permafrost thaw feedbacks?

### Characteristics of the Plant-Influenced Microbiome

Microbial communities of Stordalen mire have been well studied but previous work has focused on bulk peat samples especially at depth (Mondav et al., 2017; Monteux et al., 2018; Woodcroft et al., 2018a). This represents the first known examination of plant-associated microbial communities at this site. Our most dominant phyla matched those seen in previous work in which Proteobacteria and Acidobacteria were the most common with Bacteroidetes and Actinobacteria also common (Mondav et al., 2017). This profile matched the general profiles found in previous investigation into bulk peat communities at this site by Mondav et al. (2017) but with stronger and more consistent dominance of Proteobacteria and lower relative abundance of Acidobacteria across all habitat types, particularly in the phyllosphere. This is consistent with previous work that has shown enrichment of alpha- and beta-proteobacteria in plant-associated communities – particularly the rhizosphere – as compared to bulk soil (Knief et al., 2012; Nuccio et al., 2016). While Mondav et al. (2017) found high relative abundance of Euryarchaeota in fen samples, we saw very low numbers with the highest abundance in fen bulk peat. We found four phyla in much higher abundance than Mondav et al. (2017) (in which they were absent or comprised less than 1% of the community): Firmicutes, Cyanobacteria, Armatimonadetes, and Spirochaetes. In contrast, we saw lower than 1% abundance of the following phyla that were reported at high abundance by Mondav et al. (2017): Microgenomates, Woesearchaeota, Thaumarchaeota, and Bathyarchaeota. These differences were strongest in the plant-associated microbial communities. Thus while our overall community composition was similar to that found in examinations of the deeper soil profile, our results are consistent with the idea that there are plant-mediated differences in microbial communities in the surface soil.

Our study also found substantially lower diversity than Mondav et al. (2017), who found a mean OTU richness of 721 with a range of 309–1,226. This may be because they report total diversity in pooled samples as opposed to our singletons which may have allowed resulted in the discovery of more different taxa. Additionally, our study used amplification of the V4 region and Illumina MySeq while Mondav et al. (2017) amplified the V6–V8 region and used 454 sequencing. Different regions of the 16S gene are known to provide different degrees of taxonomic resolution (Bukin et al., 2019) as well as biases in taxonomic composition (Sinclair et al., 2015; Fischer et al., 2016). Finally, our sampling approach for bulk peat was substantially different. Because we were sampling in the rooting zone close to plants, it is possible that even our bulk peat samples were more strongly plant influenced than those of Mondav et al. (2017), which were targeted at the peat profile. Thus both sequencing differences and plant influence may account for some of the differences in diversity and community composition between our bulk peat samples and those of Mondav et al. (2017).

### Biotic Versus Environmental Filtering

Variance partitioning of relative abundances of the microbial community provided support for our hypothesis that habitat is less important for structuring phyllosphere microbial communities. In the phyllosphere, plant associate explained more variation than habitat – particularly when overlapping factors were excluded – indicating that biotic filtering was stronger than environmental filtering (Table 2). Additionally, both plant associate and habitat explained more variance in community composition in the rhizosphere than the phyllosphere. This is consistent with there being higher dispersal rates and less filtering (biotic or abiotic) on phyllosphere communities. Indeed, habitat and plant associate together only explained 20% of the variation in phyllosphere community composition (as compared to 35% in the rhizosphere and 33% in the bulk peat). This is consistent with previous findings that the phyllosphere tends to be more variable than the rhizosphere (Lebeis, 2015). We hypothesize that phyllosphere microbial communities are either more strongly determined by stochastic processes or simply more strongly impacted by variables not associated with plant-associate or thaw stage such as humidity or radiation. Testing these hypotheses was beyond the scope of the current study but would be a useful step for improving our understanding of phyllosphere microbial communities.

In contrast, in the rhizosphere plant associate and habitat explained similar amounts of variance in community composition (Table 2), indicating that both were important but neither strongly overrode the other. This was not entirely in line with our hypothesis which proposed that environmental filtering by habitat would override biotic effects in the rhizosphere. Indeed, biotic effects explained slightly more variance than habitat (8% vs 7%). This is particularly striking given the large differences in habitat type, ranging from dry palsa to fully inundated fen. This difference was large enough that our plant species did not span all three habitats, and some were found in only a single habitat. This last is a major cause of the high degree of overlap in the variance explained by both plant and habitat. While it has long been known that plants strongly influence their rhizospheres (Berg and Smalla, 2009; Knief et al., 2012; Ferrera-Rodríguez et al., 2013; Philippot et al., 2013; Lebeis, 2015; Lee et al., 2015), this influence is not always sufficient to override environmental differences (Kumar et al., 2016, 2017; Nuccio et al., 2016). Thus, it is striking that even with such a large difference between habitat types, plant associate still explained as much or slightly more variation than habitat.

One hypothesis to explain why previous studies have found that environmental factors were more important than species associate – in contrast to our finding and despite the strong influence of plants on rhizosphere communities – is that differences between plant functional groups are a key factor. Nuccio et al. (2016) found that rhizosphere community composition was most strongly correlated with moisture and then with region but not with species. However, this study was conducted within grasslands and focusing on a single plant genus (*Avena*). While our study included a strong moisture gradient, it also included a number of different plant functional types (sphagnum mosses, sedges, forbs, and ericaceous shrubs). It may be that the larger differences between plant functional types explain why plant associate had such a strong influence on rhizosphere community across our large environmental gradient. Indeed, research has shown substantial differences between plant species in quantity and quality of root litter inputs in thawing permafrost ecosystems which could influence microbial community composition (Blume-Werry et al., 2019). Cluster analysis based on relative abundances showed that sedge rhizosphere communities from the wet bog and fen habitats grouped together – though the sedge-associated community in the dry palsa grouped with its habitat (Figure 8). This is consistent with the finding of Nuccio et al. (2016) that moisture is a key factor filtering rhizosphere communities. Similarly, *Sphagnum* phyllosphere communities from palsa and bog habitats were more similar to each other than to any other communities, particularly when clustered by organisms’ network importance (Figure 8). Sphagnum mosses are known to cultivate a specialized endophytic microbial community (Bragina et al., 2012, 2014; Bragina et al., 2015; Holland-Moritz et al., 2018), however, this community has also been known to demonstrate high specificity to host and environmental factors. This means it is somewhat surprising that the phyllosphere communities from the different *Sphagnum* species and very different hydrologic conditions of the palsa and bog were so similar. Overall, these findings are consistent with the idea that environment first filters microbial community (especially through moisture), then biotic interactions with plants further shape composition.

### Keystone Organisms

The high diversity of microbial communities provides a challenge in considering how to weight each taxonomic group in terms of its importance to community composition. Typically, community composition is compared using relative abundance as a weighting, which essentially assumes that the higher abundance of an organism, the larger is its importance to the community composition. While this may be true in many cases, when considering the impacts of community changes on ecosystem function often low-abundance organisms can be highly influential (Paine, 1969; Shade et al., 2014; Banerjee et al., 2018). Network analysis provides a mechanism for identifying these keystone organisms in microbial communities. Previous studies have used high betweenness and centrality scores to identify keystone taxa within microbial communities, however, there is not yet a consensus over which network metrics best measure keystoneness (Martín González et al., 2010; Berry and Widder, 2014; Vick-Majors et al., 2014; Banerjee et al., 2016a, b). An alternate approach has been applied by Allesina and Pascual (2009) who found that PageRank scores were strongly associated with extinction cascades in food webs. This provides a strong biological rationale for use of this metric. Additionally, we found that PageRank was strongly correlated with the other metrics of betweenness and centrality (Supplementary Figure S4). Therefore, we used PageRank scores as an indication of an organism’s importance to its network and defined keystone organisms as those in the 99th percentile of importance (PageRank score) for each community (network).

Environmental filtering appeared to influence keystone organisms more strongly than the overall microbial community. Habitat explained an increased portion of the variance in microbial community across all compartments when only keystone organisms were considered (Table 2). Similarly, clustering communities based on keystoneness (PageRank score) changed the grouping of rhizosphere communities such that sedges no longer clustered together, indicating that habitat was a more critical factor in determining keystone taxa in this case (Figure 7). On the other hand, in the phyllosphere, sphagnum-associated communities clustered farther apart from others when only keystone organisms were considered, suggesting that these organisms were more strongly influenced by their plant associate. It is not surprising that some organisms will be more strongly influenced by habitat and others by plant-associate, but these findings suggest that it is important to think carefully about which metrics we use to measure changes in community composition since they may alter our results.

We found some evidence that keystone organisms can be important to C-cycling as well as community assembly processes. Five keystone OTUs belonged to functional groups of interest, one methanogen and four putative methanotrophs. The methanogen was a keystone in bulk peat and all but one of the methanotrophs were keystones in rhizosphere and/or phyllosphere communities (Supplementary Table S1). Since the effect of methanotrophs can be large enough to remove up to 50–97% of CH_4_ from an ecosystem (Segarra et al., 2015), these keystone organisms may indeed play an important role in ecosystem processes.

### Potential Impacts on CH_4_-Cycling

An important change taking place as permafrost thaws is conversion to increasingly hydric habitats with increasing methane (CH_4_) production (Johansson et al., 2006). This change has the potential to have a major impact on climate change since CH_4_ has a warming potential roughly 25 times that of CO_2_ (IPCC, 2013). In order to understand the implications of biotic and abiotic filtering of microbial communities on C-cycling in these systems we separately investigated organisms associated with CH_4_ cycling. Use of 16S data to infer functionality carries with it caveats since any inferences are based solely on taxonomic associations with organisms known to perform particular functions. However, complex traits such as methanogenesis and methanotrophy tend to be more highly conserved than other microbial traits, with methanogenesis in particular conserved across entire families or even orders (Martiny et al., 2015). Methanotrophy is also generally conserved within families, though there are examples where it has been lost by particular species (Tamas et al., 2014). While activity of an organism or any particular gene cannot be inferred from presence alone, previous research at this site has used metagenomic and metatranscriptomic data to show the presence and activity of the families identified here as putative methane-cycling organisms (Mondav et al., 2014; Singleton et al., 2018; Woodcroft et al., 2018b).

Putative methanotrophs identified in this dataset were members of groups previously identified as encoding genes for methanotrophy at this site (Singleton et al., 2018): Alpha-proteobacterial families Methylocystaceae, Beijerinckiaceae, Hyphomicrobiaceae, and Methylocystaceae; Gammaproteobacterial families Methylococcaceae and Crenotrichaceae; and the Verrucomicrobia order Methylacidi philales (Smith and Wrighton, 2019). We found putative methanotrophs across all habitats, plants, and compartments. This is perhaps unsurprising in an environment with high CH_4_ production. The most ubiquitous were members of Methylocystaceae, and this family also reached the highest relative abundances, which were in the phyllosphere – particularly when associated with *A. polifolia* and sedges (*Eriophorum* spp. and *C. rotundata*). In the palsa and fen, Methylacidiphilales and Crenotrichaceae, respectively, were also very common. Variation in methanotroph communities was driven by both habitat and plant associate, with the latter more important in the phyllosphere and the former more important in the rhizosphere and peat (Table 3). This suggests that methanotrophs in the rhizosphere are more influenced by soil parameters such as moisture and oxygenation, whereas methanotrophs in the phyllosphere are shaped by plant characteristics such as structure or biochemistry. If so, future investigations of functional gene abundance and activity may identify associations between plant functional types and microbial functional types. For instance, sedges have aerenchymous roots that may vent methane to the phyllosphere (Iversen et al., 2015) thereby shaping the microbial community.

Putative methanogens at this site were all members of genuses previously identified as methanogens at this site based on functional genes (Woodcroft et al., 2018a) belonging to Methanobacteriaceae (Methanobacterium), Methanoregulaceae (Candidatus Methanoregula), and Methanosarcinaceae (Methanosarcina). These organisms were distributed across all habitats and compartments, though they were rare in the phyllosphere and most abundant in the bulk soil (Figure 5b). Although methanogenesis is an anaerobic process, methanogens and even methane production have been previously observed in bulk aerobic environments such as the phyllosphere (Keppler et al., 2006; Taffner et al., 2019), and it is possible they inhabited anaerobic pockets of leaf tissue. Plant associate explained much more variation in relative abundances of putative methanogens in the rhizosphere than it did for any other group of organisms (Table 4). Tighter interaction between plant roots and putative methanogens than other groups could be driven by differences in substrate availability due to root exudates or differences in electron acceptor availability as a result of gas transport by roots. This strong influence of plant associate on explaining putative methanogen abundance has important implications for climate change since CH_4_ production from thawing permafrost ecosystems is a major potential feedback to global warming.

## Conclusion

We began by asking to what degree plant community composition versus environmental shifts are important to structuring microbial communities under climate change. We additionally asked whether the relative contribution of each differs when considering only keystone organisms or particular functional groups versus the whole microbiome. Such questions are important because microbes are responsible for driving many important processes, such as C-cycling, which are changing rapidly with climate. If we can better understand which forces structure microbial communities or key functional groups, we can better predict the implications of changing conditions for these processes. Our results showed that such prediction is difficult, since much of the variation in microbial community composition remains unexplained by either biotic (plant) or environmental filtering. This may be an argument for investigating the role of more specific environmental variables such as moisture or pH in driving microbial community composition, but it should be noted that when investigating more specific drivers it becomes more likely that their effects will vary amongst microbial groups. We did show that both types of filtering play a role, with their relative importance differing between rhizosphere and phyllosphere and for particular functional groups but not for keystone organisms. Putative methane-cycling microbes were especially strongly impacted by plant associates beyond the effects of ecosystem transition, suggesting that plant community composition may be particularly important to shifts in the CH_4_ cycle. Further research should confirm the functionality and quantify activity levels of these putative CH_4_-cycling organisms when associated with different plant species. Such information may prove particularly useful to remote-sensing approaches, which could potentially use maps of plant community composition to infer information about CH_4_-cycling microbes. However, such approaches should consider microbial functional groups individually, rather than entire microbiomes, since the importance of plant versus environment seems to vary by group.

## Data Availability Statement

The datasets generated for this study can be found in the IsoGenie Database: https://isogenie-db.asc.ohio-state.edu/, National Center for Biotechnology Information Sequence Read Archive, and Study Accession: PRJNA599435.

## Author Contributions

AM, MH, VK-C, and VR designed the study in consultation with ED. AM and MH conducted fieldwork. AM and VK-C performed the lab work. AM, VK-C, and MH performed data processing, analyses, and generated figures. BB performed network analyses, including generation of associated figures and text. AM, MH, VK-C, VR, and SS secured funding for this work. All authors participated in manuscript preparation.

## Conflict of Interest

The authors declare that the research was conducted in the absence of any commercial or financial relationships that could be construed as a potential conflict of interest.
