# Supplementary material for: Biotic and Environmental Drivers of Plant Microbiomes Across a Permafrost Thaw Gradient
Source: Front Microbiol. 2020 May 15;11:796. doi: 10.3389/fmicb.2020.00796 (PMC7243355; doi:10.3389/fmicb.2020.00796)

**Supplemental Tables**

**Table S1:** Taxonomy of keystone OTUs (those in the 99^th^ percentile of importance for a network and networks in which they attained this level of importance).

**Table S2:** Number of keystone OTUs found in each network type

|  |  | **Phyllosphere** | **Rhizosphere** | **Peat** |
| --- | --- | --- | --- | --- |
| **Palsa** | *A. polifolia* | 2 | 4 | 3 |
|  | *R. chamaemorus* | 3 | - | 4 |
|  | *E. vaginatum* | 2 | 4 | 5 |
|  | *Sphagnum sp* | 3 | 4 | - |
| **Bog** | *A. polifolia* | 4 | 5 | 5 |
|  | *C. rotundata* | 2 | 5 | 6 |
|  | *E. vaginatum* | 2 | 5 | 7 |
|  | *Sphagnum sp* | 2 | 5 | - |
| **Fen** | *C. rotundata* | 3 | 5 | 6 |
|  | *E. angustifolium* | 3 | 6 | 5 |

**Table S3:** Putative methanotrophs

| **Domain** | **Phylum** | **Class** | **Order** | **Family** | **Genus** |
| --- | --- | --- | --- | --- | --- |
| Bacteria | Proteobacteria | Alphaproteobacteria | Rhizobiales | Beijerinckiaceae | Other |
| Bacteria | Proteobacteria | Alphaproteobacteria | Rhizobiales | Beijerinckiaceae | Beijerinckia |
| Bacteria | Proteobacteria | Alphaproteobacteria | Rhizobiales | Hyphomicrobiaceae | unknown |
| Bacteria | Proteobacteria | Alphaproteobacteria | Rhizobiales | Hyphomicrobiaceae | Devosia |
| Bacteria | Proteobacteria | Alphaproteobacteria | Rhizobiales | Hyphomicrobiaceae | Hyphomicrobium |
| Bacteria | Proteobacteria | Alphaproteobacteria | Rhizobiales | Hyphomicrobiaceae | Rhodoplanes |
| Bacteria | Proteobacteria | Alphaproteobacteria | Rhizobiales | Methylocystaceae | Other |
| Bacteria | Proteobacteria | Alphaproteobacteria | Rhizobiales | Methylocystaceae | unknown |
| Bacteria | Proteobacteria | Alphaproteobacteria | Rhizobiales | Methylocystaceae | Methylopila |
| Bacteria | Proteobacteria | Alphaproteobacteria | Rhizobiales | Methylocystaceae | Methylosinus |
| Bacteria | Proteobacteria | Alphaproteobacteria | Rhizobiales | Methylocystaceae | Pleomorphomonas |
| Bacteria | Proteobacteria | Alphaproteobacteria | Rhizobiales | Methylocystaceae | Rhodoblastus |
| Bacteria | Proteobacteria | Gammaproteobacteria | Methylococcales | Crenotrichaceae | Crenothrix |
| Bacteria | Proteobacteria | Gammaproteobacteria | Methylococcales | Methylococcaceae | Other |
| Bacteria | Proteobacteria | Gammaproteobacteria | Methylococcales | Methylococcaceae | unknown |
| Bacteria | Proteobacteria | Gammaproteobacteria | Methylococcales | Methylococcaceae | Methylomonas |
| Bacteria | Verrucomicrobia | [Methylacidiphilae] | Methylacidiphilales | unknown | unknown |

**Table S4:** Putative methanogens

| **Domain** | **Phylum** | **Class** | **Order** | **Family** | **Genus** |
| --- | --- | --- | --- | --- | --- |
| Archaea | Euryarchaeota | Methanobacteria | Methanobacteriales | Methanobacteriaceae | Methanobacterium |
| Archaea | Euryarchaeota | Methanomicrobia | Methanomicrobiales | Methanoregulaceae | Candidatus Methanoregula |
| Archaea | Euryarchaeota | Methanomicrobia | Methanosarcinales | Methanosarcinaceae | Methanosarcina |

**Supplemental Figures**

**Figure S1: Rarefaction Curves**

**
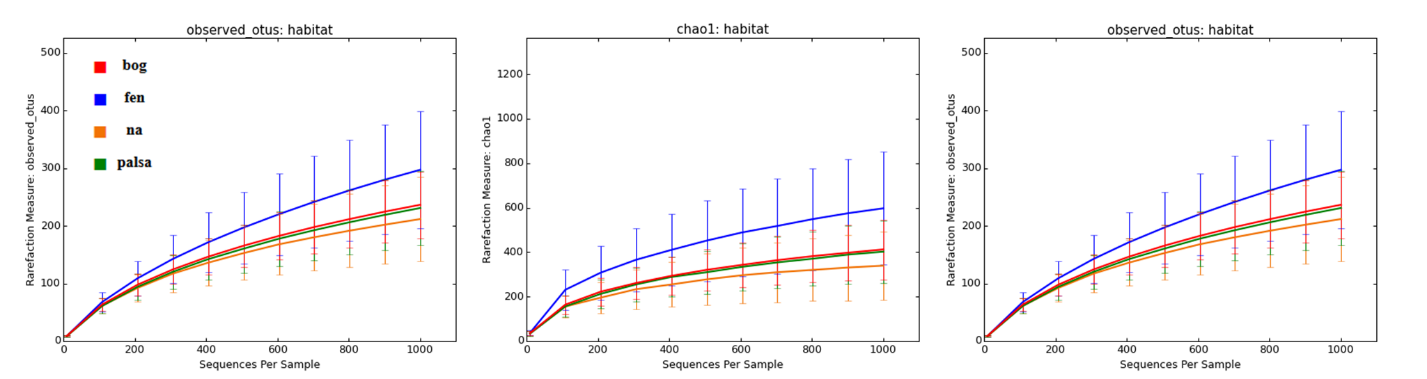
**

**
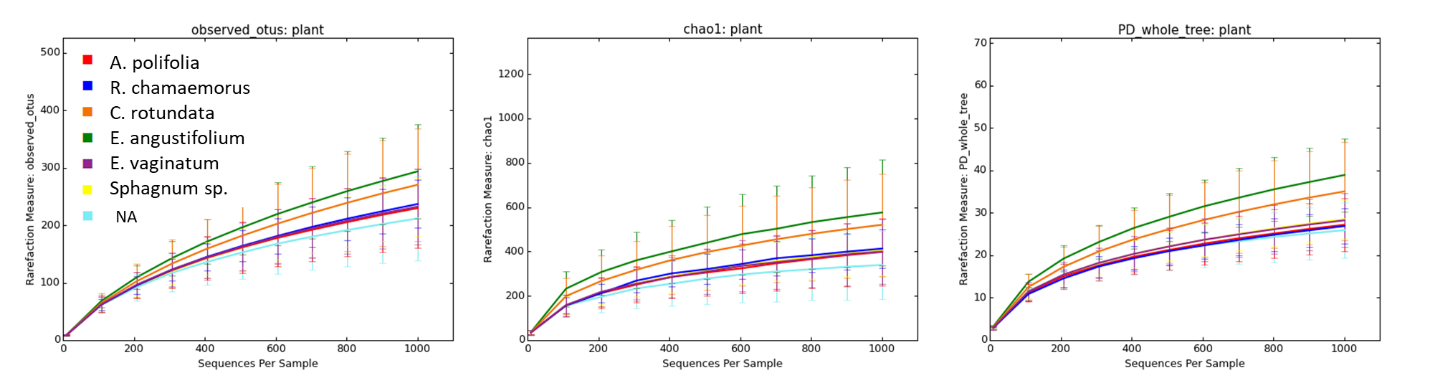
**

**
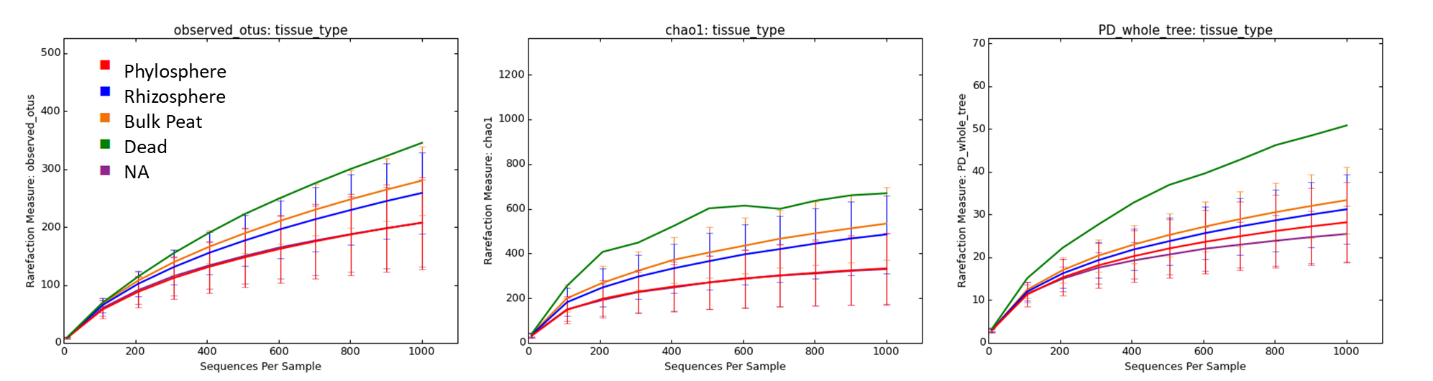
**

**Figure S2: Phyla enriched in each habitat (LDA>2) for each compartment based on Lefse analysis**

Phyllosphere


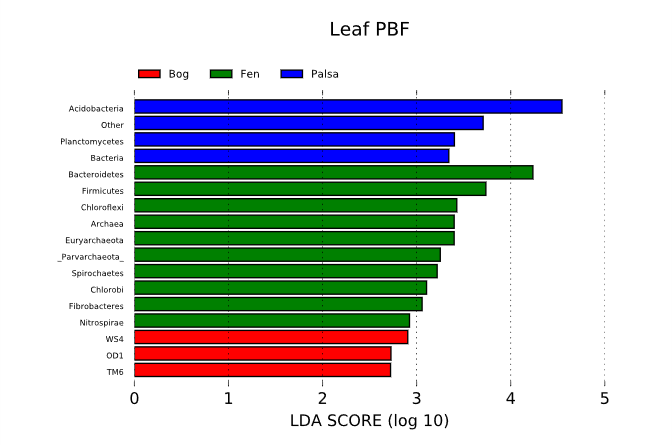


Rhizoosphere


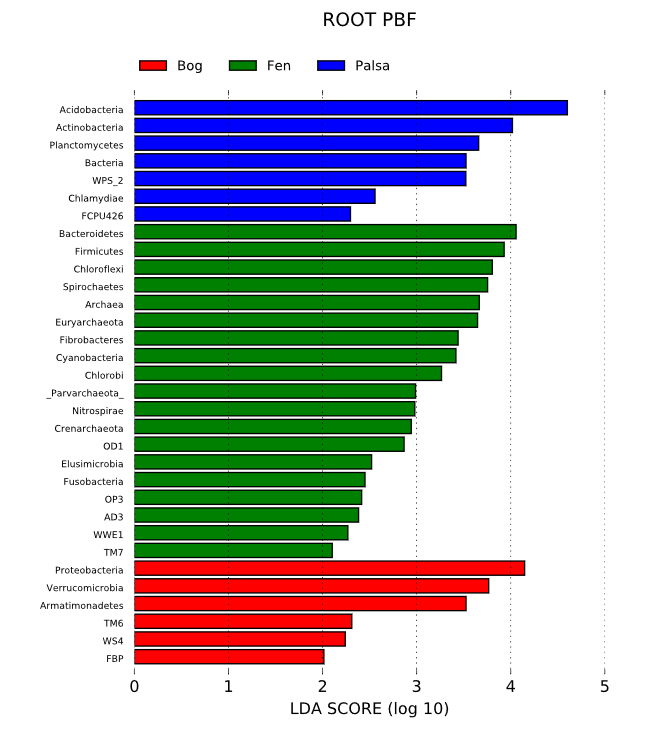


Bulk Peat


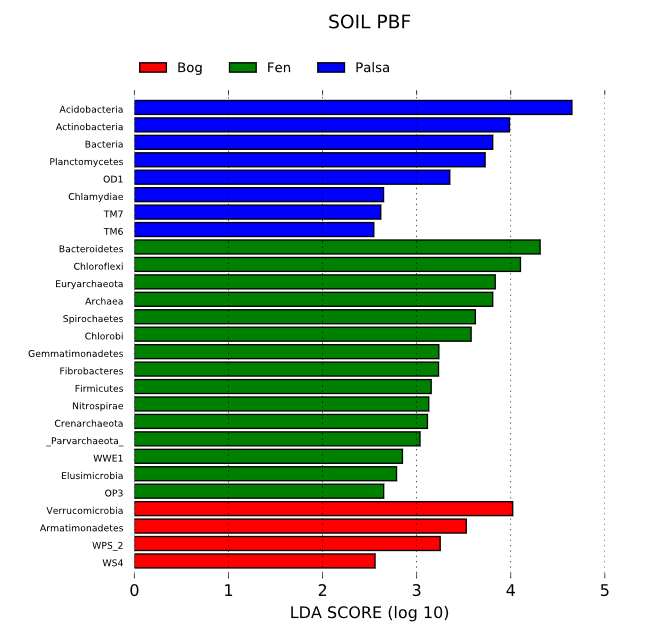


**Figure S3: Hierarchical Clustering based on Jaccard Dissimilarity**

**
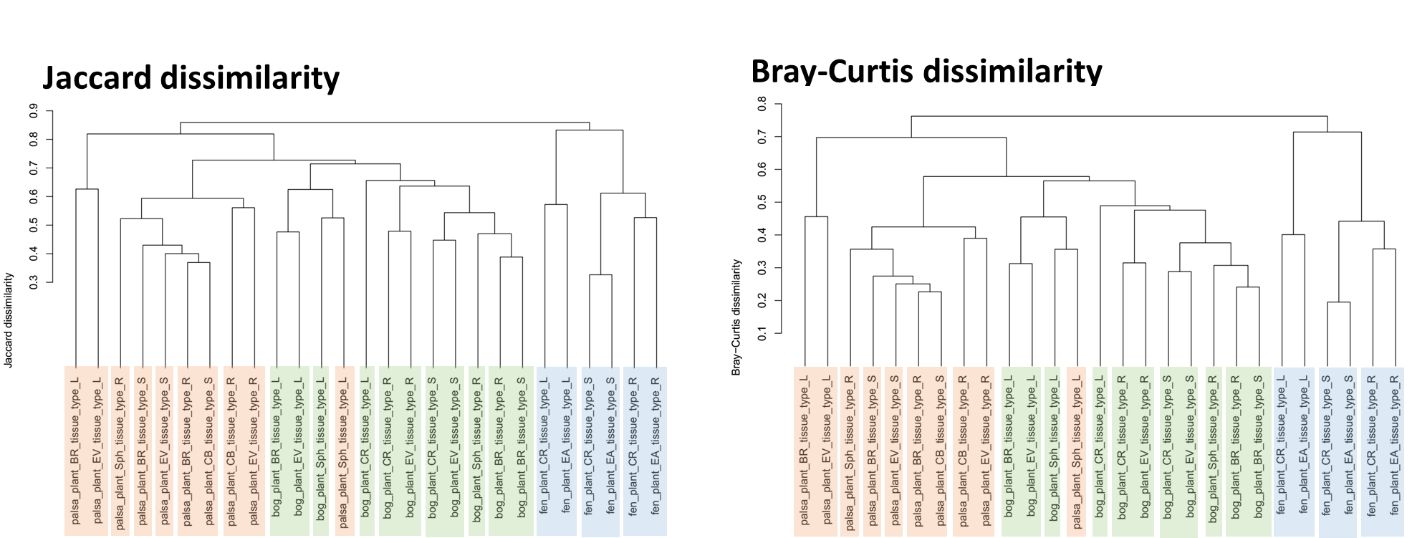
**

**Codes:**

L= phyllosphere

R= rhizosphere

S= bulk peat

BR= *A. polifolia*

EV= *E. vaginatum*

Sph= *Sphagnum*

CB= *R. chamaemorus*

CR= *C. rotundata*

EA= *E. angustifolium*

**Figure S4: Relationship between PageRank vs other metrics of Betweenness and Centrality**

**
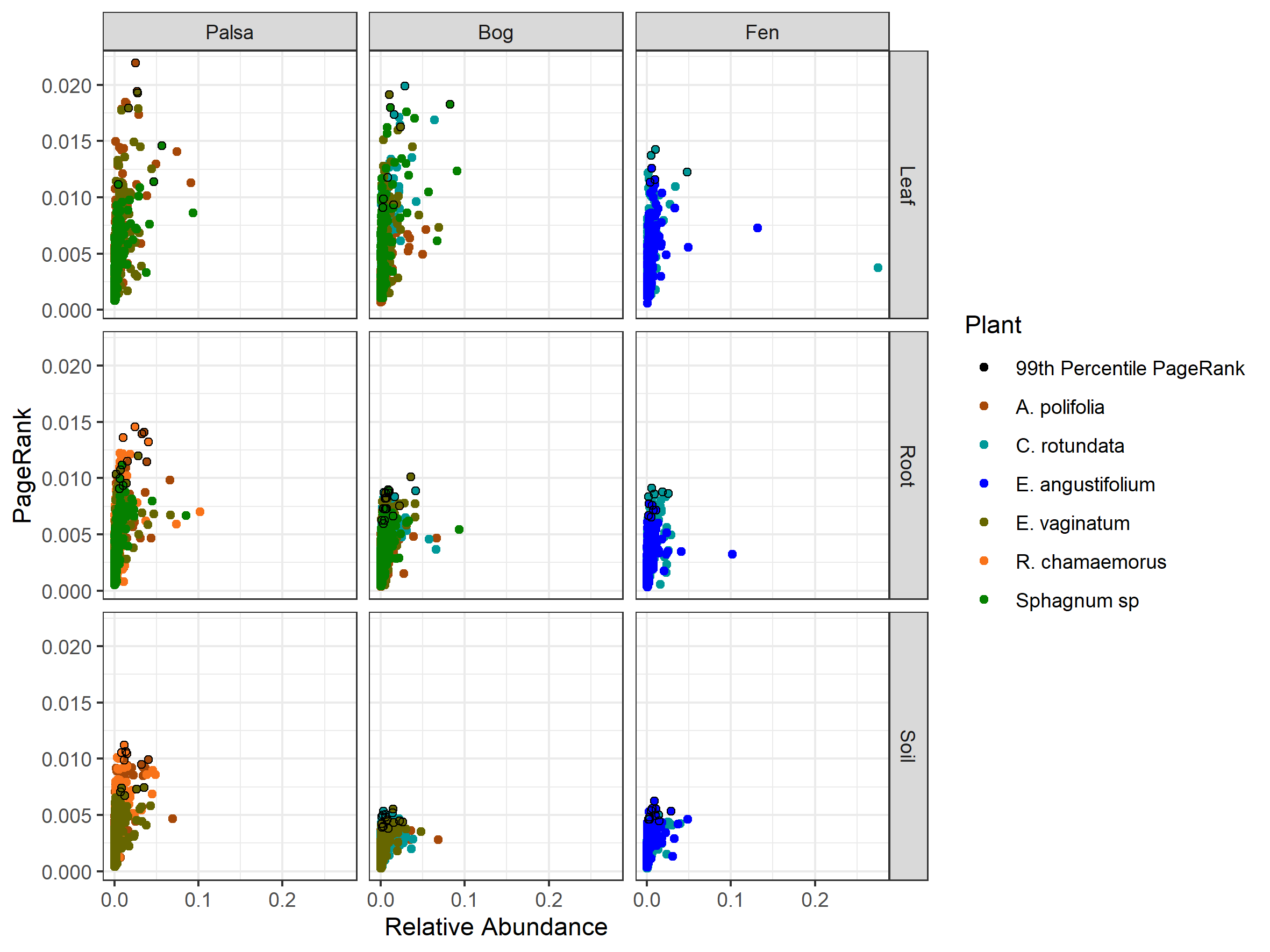
**

**
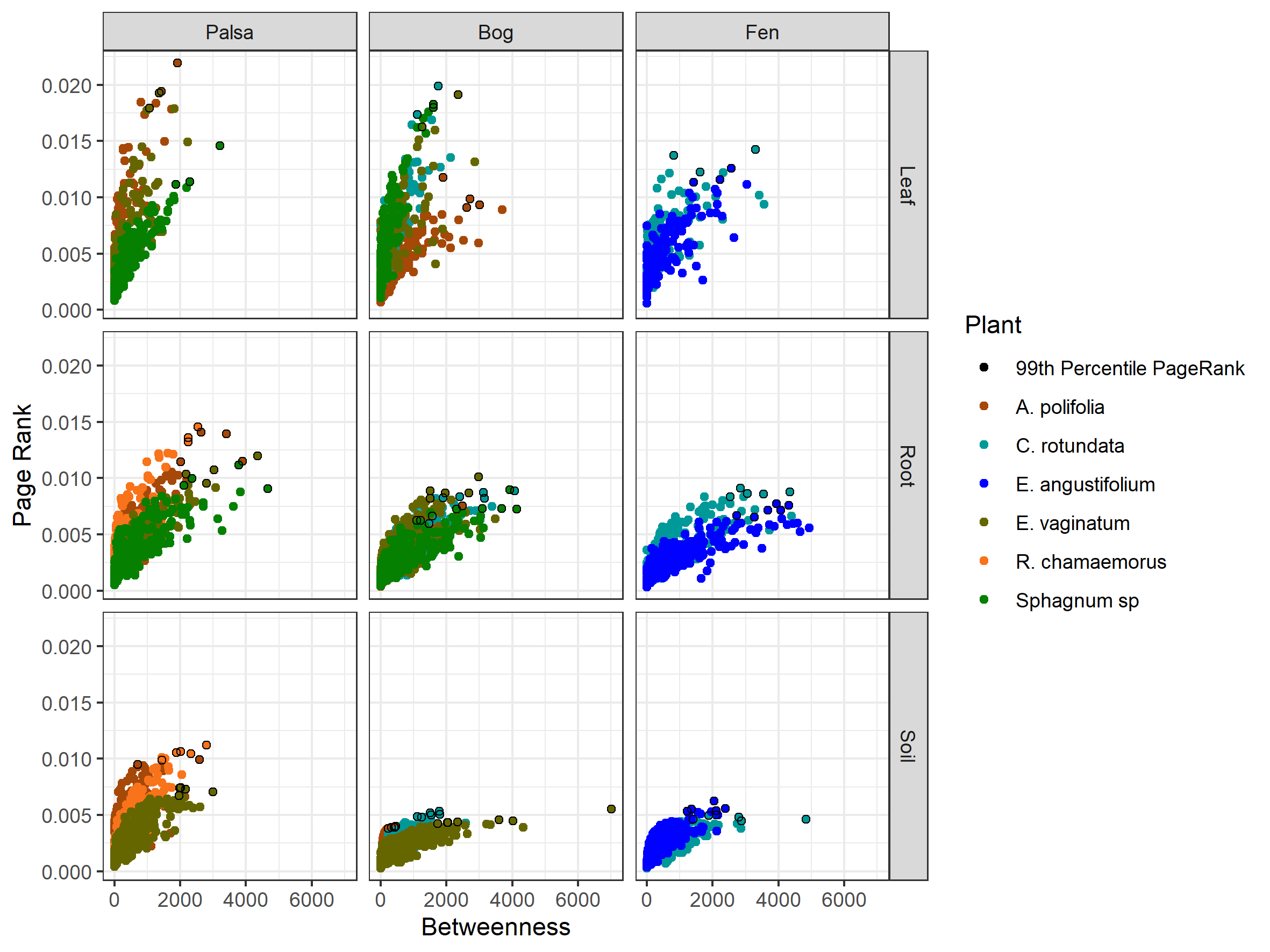
**

**
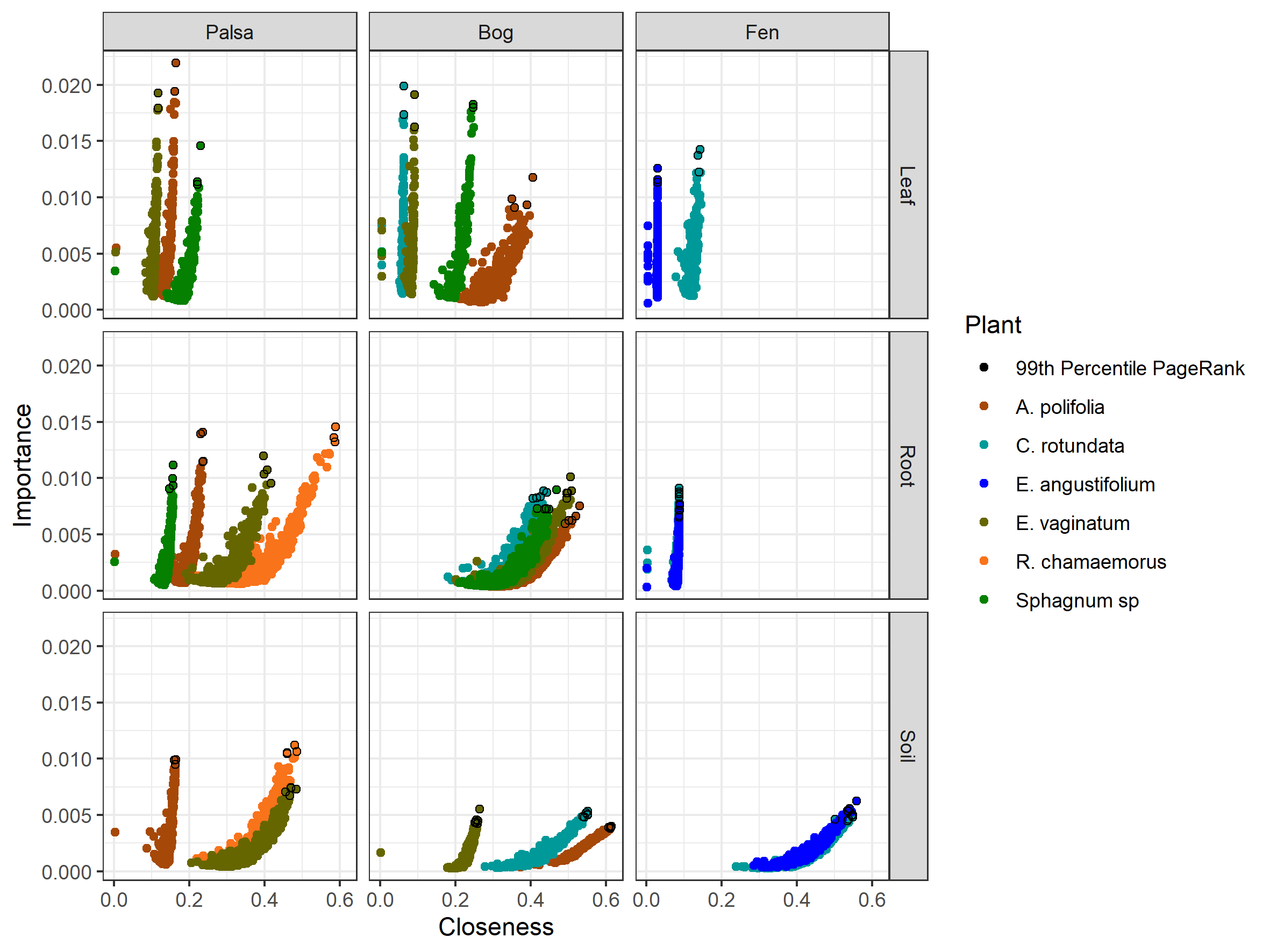
**

**
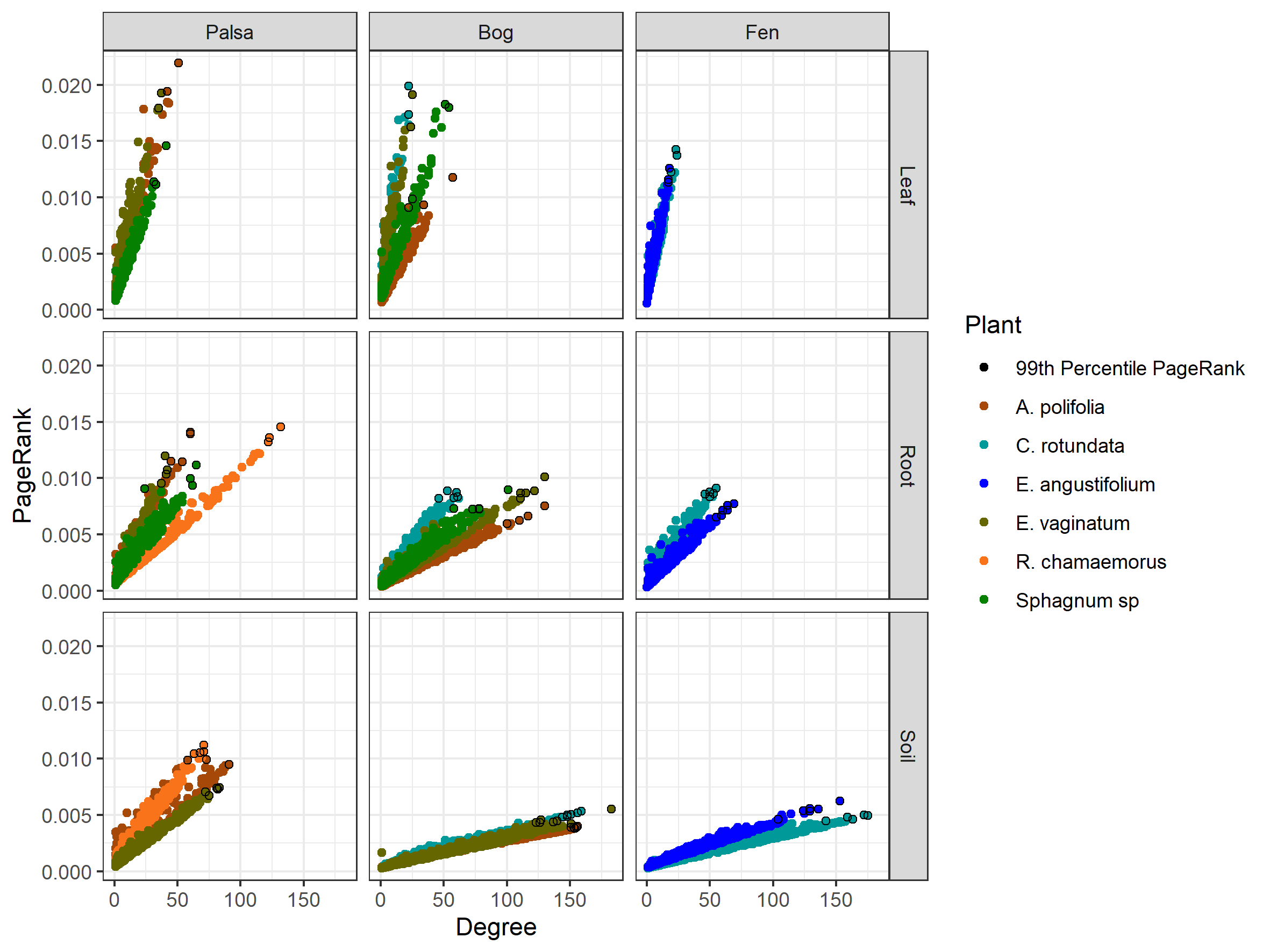
**

**
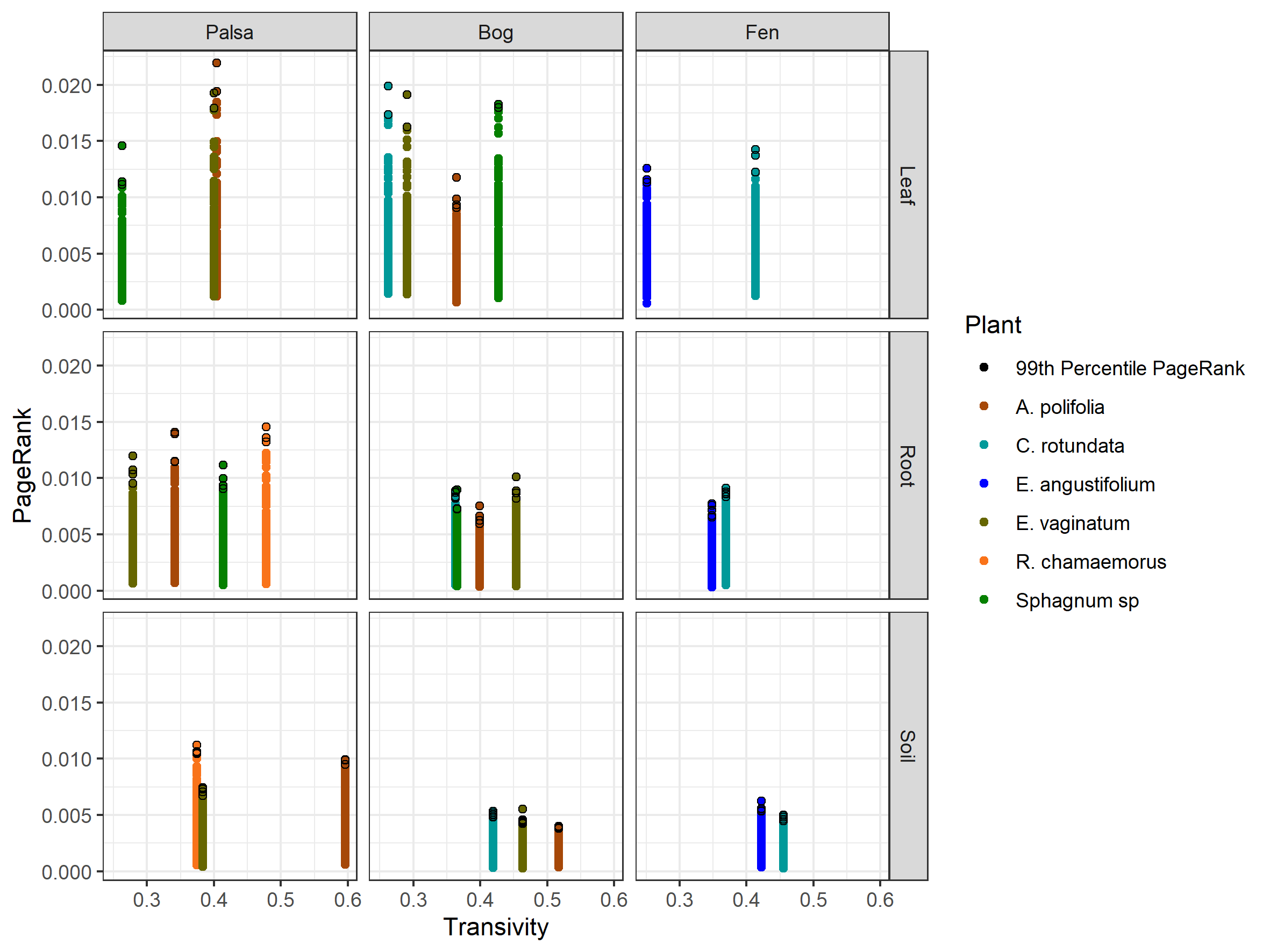
**

**Fig S5: Correlation between PageRank and other measures within each network. Note that the variance for correlations with transitivity was zero so it is not included here.**


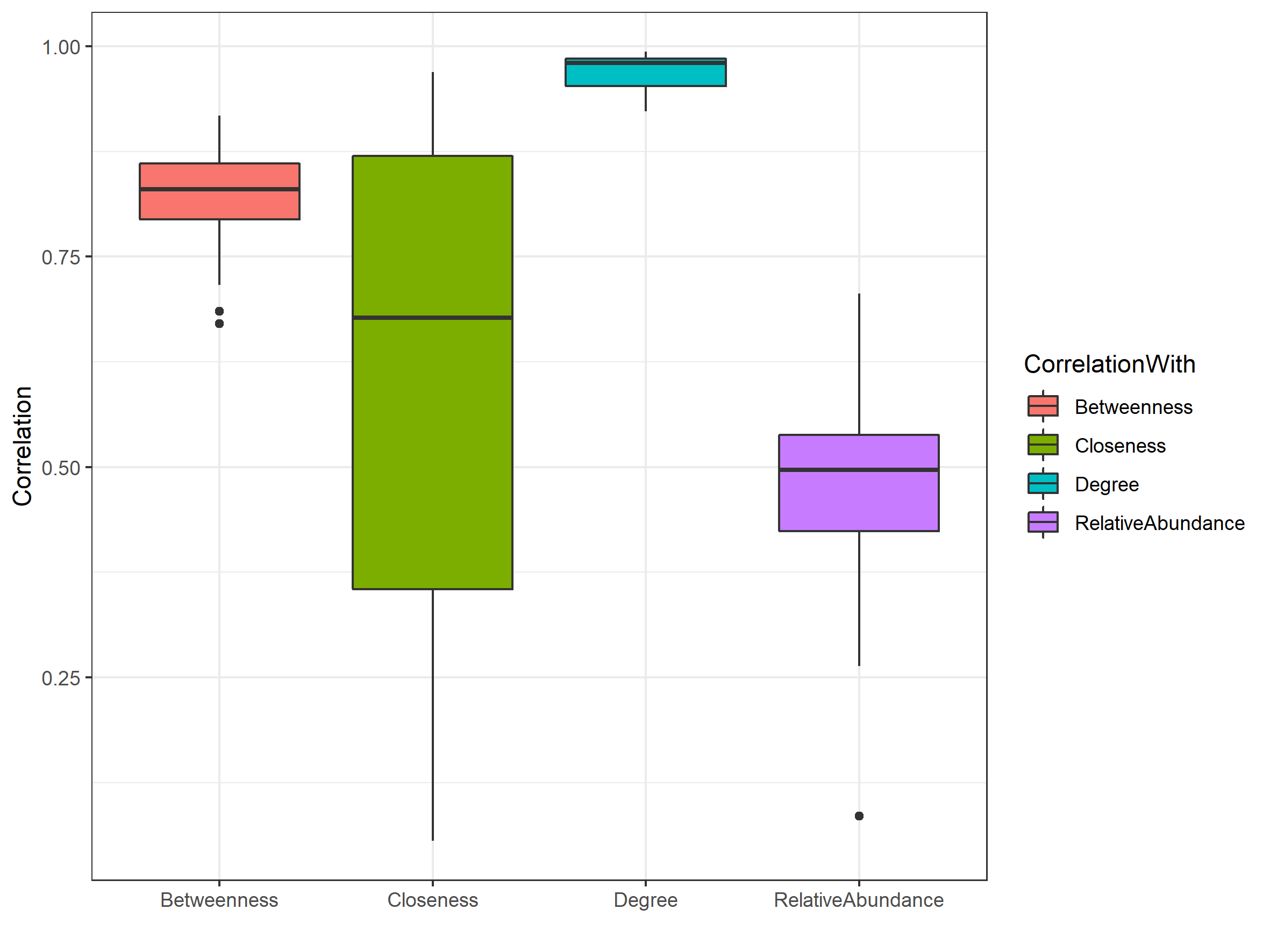

Supplement: Supplementary file 1 [file Table_1.DOCX]
